# Gait Analysis in a *Mecp2* Knockout Mouse Model of Rett Syndrome Reveals Early-Onset and Progressive Motor Deficits

**DOI:** 10.1371/journal.pone.0112889

**Published:** 2014-11-13

**Authors:** Kamal K. E. Gadalla, Paul D. Ross, John S. Riddell, Mark E. S. Bailey, Stuart R. Cobb

**Affiliations:** 1 Institute of Neuroscience and Psychology, College of Medical, Veterinary and Life Sciences, University of Glasgow, Glasgow, United Kingdom; 2 School of Life Sciences, College of Medical, Veterinary and Life Sciences, University of Glasgow, Glasgow, United Kingdom; 3 Pharmacology Department, Faculty of Medicine, Tanta University, Egypt; University of Insubria, Italy

## Abstract

Rett syndrome (RTT) is a genetic disorder characterized by a range of features including cognitive impairment, gait abnormalities and a reduction in purposeful hand skills. Mice harbouring knockout mutations in the *Mecp2* gene display many RTT-like characteristics and are central to efforts to find novel therapies for the disorder. As hand stereotypies and gait abnormalities constitute major diagnostic criteria in RTT, it is clear that motor and gait-related phenotypes will be of importance in assessing preclinical therapeutic outcomes. We therefore aimed to assess gait properties over the prodromal phase in a functional knockout mouse model of RTT. In male *Mecp2* knockout mice, we observed alterations in stride, coordination and balance parameters at 4 weeks of age, before the onset of other overt phenotypic changes as revealed by observational scoring. These data suggest that gait measures may be used as a robust and early marker of MeCP2-dysfunction in future preclinical therapeutic studies.

## Introduction

Rett Syndrome (RTT) is an X-linked disorder caused by loss-of-function mutations in the *MECP2* gene [1] and affecting ∼1 in 10,000 females. The RTT phenotype is characterised by a constellation of typical and associated features [2], most of which involve brain dysfunction, with notable characteristic features being developmental regression, loss of motor and language skills and stereotyped movements of the hands. Gait abnormalities and motor dysfunction are among the main criteria for diagnosis of Rett Syndrome [2]. Subsequent to the regression phase, most RTT patients gain or regain only modest locomotor skills, most being confined to wheelchairs or requiring lifelong assistance with walking. Gait has been analysed in mobile RTT patients post-regression and has been reported to be wide-based and clumsy [3]. Many of the features of RTT are successfully modelled in *Mecp2* knockout mice, and locomotor behaviour has been shown to be affected [4]–[7]. Although RTT has commonly been thought to be neurodevelopmental in character, recent work has demonstrated that the phenotype can be rescued to an appreciable extent by restoration of *Mecp2* gene function at any stage postnatally [7], [8] whilst adult inactivation of *Mecp2* results in a RTT-like phenotype [9], [10]. These studies suggest that RTT may potentially be both preventable and reversible in patients. As more therapeutic avenues are explored in RTT, the characterisation of good outcome measures for therapeutic interventions is becoming of prime importance.

Previous gait-related studies of *Mecp2* knockout mice have utilised either paw inking or video imaging on a static surface [7], [11], [12] and have reported significant differences in several gait parameters in symptomatic animals. However, these approaches are limited in the range of gait phenotypes that can be characterised in detail, and did not account for speed differences between the *Mecp2* knockout mice (which have clear motor and movement deficits) and wild-type controls. Moreover, there is little data concerning the longitudinal trajectory of the gait-related phenotype in these mice, which is important for the development of outcome biomarkers for use in pre-clinical therapeutics studies. We therefore carried out a thoroughgoing analysis, using a treadmill apparatus, of the gait-related aspects of phenotype in *Mecp2* knockout mice to inform future studies aimed at developing accurate and easy to implement outcome measures for pre-clinical therapeutic strategy testing. Because other variables in these knockout mice, such as running speed and motivation, could be confounding in a study of gait, we employed a treadmill, which allowed us to minimise any such confounding factors. Furthermore, the automated analysis provides an objective method of calculating gait parameters. We detected a number of novel gait parameters affected in knockout mice, several of which were detectable before the onset of other overt RTT phenotypes.

## Methods

### *Mecp2-stop* mice

A breeding colony of *Mecp2-stop* mice (*Mecp2* ^tm2Bird^, Jackson Laboratories Stock No. 006849) was set up using *Mecp2^stop/+^* heterozygous female stock mice (a kind gift from Prof. A. Bird, University of Edinburgh, UK). The disease-causing mutation in this RTT model is the insertion of a targeted STOP cassette upstream of exon 3, which leads to a null product from the mutant allele and is therefore akin to a gene knockout. All mice used in experiments were male *Mecp2^stop/y^* offspring resulting from a breeding scheme involving at least ten generations of backcross from a congenic C57BL/6 background onto a BALB/c background. Genotype was determined from ear clipping samples by PCR as described [8]. Mice were maintained on a 12 h light/dark cycle and provided with food and water *ad libitum*. Experiments were carried out in accordance with the European Communities Council Directive (86/609/EEC) and a project licence and approval under the UK Scientific Procedures Act (1986). The work was also approved by the University of Glasgow Ethical Review Panel.

### Phenotype Scoring

From the age of 3 weeks (weaning), mice were scored weekly for signs of the *Mecp2-stop* phenotype using an observational severity score described previously [8], [13]–[15]. Briefly, each of six observable features (mobility, gait, hindlimb clasping, tremor, breathing and general condition) were scored on a 0–2 scale (0 - no signs, 1 - mild signs; 2 - severe/obvious signs; see [8] for details), with the observer blind to genotype. Scores were aggregated to give an overall severity score out of 12 for each individual mouse.

### Gait analysis

**Figure 1:**
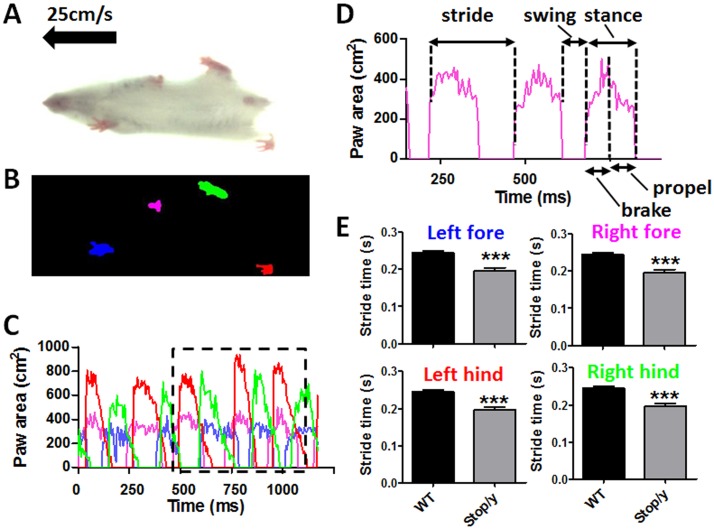
Treadmill-based measurement of gait properties in *Mecp2^stop/y^* mice. (A) Video still image showing mouse walking on transparent treadmill (viewed from below) at 25 cm/s. (B) Placement of each paw is detected from the video illustrated in (A). (C) Graph of paw area in contact with the treadmill surface for each paw over time (one session, one animal); the area outlined in black has been replotted in (D). (D) Excerpt from (C) showing calculated paw area in contact with treadmill surface over time for a representative single paw (right fore; pink in B and C), from which multiple stride indices can be obtained (labeled). (E) Column plots showing stride time measures (mean +/− SEM) for each limb in 8 week old wild-type (WT) and *Mecp2^stop/y^* mice (***p<0.001 relative to WT; *t*-test, n = 5 per genotype).

Gait was analysed using the DigiGait imaging system (Mouse Specifics Inc., Boston, MA, USA). A range of running speeds were tested in a pilot experiment to define a highest speed that all mice were able to run at, thus excluding differences in self-selected speeds as the most critical confounding factor in the interpretation of over-ground walking subjects. Digital videos of the ventral surface of the mice were captured as they ran at 25 cm/s (a speed at which all mice including the *Mecp2* knockout mice were able to steadily run for a minimum of 6 complete strides) on a transparent motorised treadmill (figure 1A). To ensure mice remained within the viewing field of the camera they were contained within an open-bottomed Plexiglas chamber with adjustable bumpers at either end. Videos captured by the camera were analysed with the supplied proprietary DigiGait software to produce digitised and vectorised paw images (figure 1B); this enabled tracking over time and the production of a waveform pattern representing the stepping cycles for each paw (figure 1C, D), from which the DigiGait software calculates numerous gait parameters/indices. Animals that refused or were unable to perform (in the case of some *Mecp2^stop/y^* mice) were removed from the study.

### Statistical analysis

All results are given as mean ± standard error of the mean (SEM). Groups were compared using 2-way repeated measures ANOVA with Tukey *post hoc* pairwise comparisons, or the Mann Whitney test, as appropriate (Minitab 16.0, Minitab Inc., USA).

## Results

**Figure 2:**
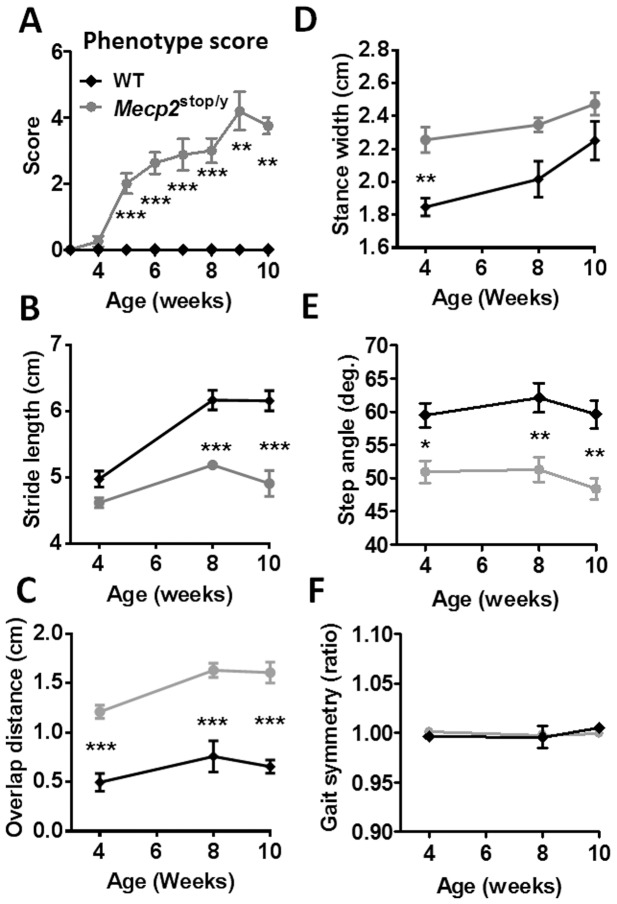
Automated gait analysis reveals early signs of motor defects in *Mecp2^stop/y^* mice. (A) Plot showing aggregate phenotype severity score (mean +/− SEM) in male wild-type (WT, black diamond symbols) and *Mecp2^stop/y^* (grey circles) mice. *Mecp2^stop/y^* mice show a gradual phenotype score progression that is significantly different from WT (WT n = 7; *Mecp2^stop/y^* n = 8 at 3–7 weeks, n = 7 at week 8, n = 5 at week 9 and n = 4 at week 10); decreasing numbers of *Mecp2^stop/y^* mice are explained by early death of some mutant mice. (B–F) Plots showing stride length, overlap distance, stance width, step angle and gait symmetry (mean +/− SEM) measured in WT and *Mecp2^stop/y^* mice at 4, 8 and 10 weeks (WT n = 7 at 4 weeks and 5 at 8 & 10 weeks; *Mecp2^stop/y^* n = 6 at 4 weeks, n = 5 at weeks 8 & 10. The treadmill speed was 25 cm/s. *p<0.05 **p<0.01 ***p<0.001, Mann Whitney pairwise comparisons (A) and 2-way repeated measures ANOVA with Tukey *post hoc* pairwise comparisons (B–F).

The mutant phenotype trajectory was assessed by weekly observational scoring from week three onwards. From five weeks of age *Mecp2^stop/y^* animals showed a progressively more severe phenotype (figure 2A) that was significantly different from that for WT animals, which scored zero for the duration of the study (*p*<0.002 from 5 weeks onwards, Mann Whitney pairwise comparisons; we set *p*<0.01 as our alpha value for significance to control for multiple testing). *Mecp2^stop/y^* mice have reduced survival [8], [13] and consequently the study was restricted to 10 weeks.

### Subtle changes in gait parameters precede the onset of overt RTT-like signs in *Mecp2^stop/y^* mice

Treadmill-based gait analysis was used to characterise the gait of male hemizygous *Mecp2^stop/y^* mice at three different time points after birth, four weeks, eight weeks and ten weeks. Several aspects of gait were assessed, including stride, coordination and balance parameters. Stride length, the distance between initial contacts of the same paw in one complete stride, is a basic gait feature and a reduction in this parameter is commonly seen in animal models of human disease with a prominent motor component such as Parkinson disease [16]. There was a highly significant effect of genotype on stride length (F_1,32_ = 63.9, *p*<0.0001, 2-way repeated measures ANOVA). No difference was observed between *Mecp2^stop/y^* and WT animals at four weeks (figure 2B), but there were highly significant differences between genotypes at both eight weeks and ten weeks, with *Mecp2^stop/y^* animals having a 22±1.3% reduction in stride length compared to WT at 10 weeks (*p*<0.0001 Tukey *post hoc* pairwise comparisons).

Gait coordination was assessed through measurement of parameters including overlap distance, stance width, step angle and gait symmetry. During normal gait in quadrupeds such as mice the forelimbs and hindlimbs show a stereotypical pattern of movement relative to one another. The hindlimbs are typically swung forwards such that hindpaws are plantar placed during stance, close to, but often a little ahead of, the position occupied by the forepaws during the preceding forepaw stance phase. The hindpaw stride therefore overlaps that of the forepaw stride in the direction of movement (antero-posterior axis). Injury or disease can lead to a change in the spatial relationship between forelimb and hindlimb strides such that the degree of overlap between the forepaw and subsequent hindpaw stance positions may increase or decrease. For example in models of cerebellar dysfunction, an increase in overlap distance (the distance between placement of front and hind paws) is observed [17]. In the current study, *Mecp2^stop/y^* mice showed much greater overlap distance than WT mice at all time points (figure 2C, F_1,32_ = 111.42, *p*<0.001, 2-way repeated measures ANOVA with Tukey *post hoc* pairwise comparisons), even at four weeks, before an overt phenotype is observed using the observational scoring system (figure 2A). It is known that mice with lesions or diseases that affect balance and postural stability tend to adopt a wider stance, presumably to compensate for centre of gravity shifts during locomotion. Stance width (distance between the centre of the two hind paws) was significantly greater in *Mecp2^stop/y^* mice than WT at 4 weeks but there was no significant difference at 8 and 10 weeks (figure 2D, F_1,32_ = 23.79, *p*<0.001, *p*<0.01 at 4 weeks, and *p*>0.05 at 8 and 10 weeks, 2-way repeated measures ANOVA with Tukey *post hoc* pairwise comparisons). Step angle records the combined angle formed by the long axes of the hind paws in relation to the long axis of the body and reflects changes in paw rotation that are a measure of spinal dysfunction [18]. *Mecp2^stop/y^* animals showed a significantly lower step angle than WT at all time points (figure 2E, F_1,32_ = 43.23, *p<0.001*, *p* = 0.02 at 4 weeks, and *p*<0.01 at 8 and 10 weeks, 2-way repeated measures ANOVA with Tukey *post hoc* pairwise comparisons), indicating an inward rotation of the hind paws in the mutant animals. Other aspects of brain and spinal pathway-based motor coordination were assessed using the gait symmetry parameter, which measures how often strides are taken by the hindpaws relative to the forepaws. In normal gait there will be one forepaw step for every hind paw step taken. There was no difference in gait symmetry observed between the genotypes (figure 2F).

## Discussion

In this study, we used a treadmill system to investigate potential gait abnormalities in a mouse model of Rett syndrome. We aimed to characterize the onset and progression of gait phenotypes and also to evaluate whether gait abnormalities provide rapid, robust and objective outcome measures that might be utilized in future studies to assess the effectiveness of therapeutic interventions. We found that *Mecp2^stop/y^* mice showed several highly significant gait alterations including increased stride frequency, less precise paw placement and a wider gait. Gait abnormalities and gait dysfunction are key diagnostic characteristics of RTT in girls and these features represent prominent and disabling aspects of the disorder [2], [19]–[21]. *Mecp2* knockout mice have been reported previously to display gait abnormalities at the age of 5–8 weeks [7], [11], [12], and our findings agree with these reports. However, our findings reveal locomotor deficits in these mice across a wide range of gait domains including stride features and coordination, and that these deficits are evident from 4 weeks of age in the *Mecp2^stop/y^* model and are maintained over early adult life in many cases.

Early detection of biomarkers of the phenotype is potentially important in preclinical therapeutic studies. At 4 weeks of age, hemizygous *Mecp2* knockout mice are typically considered to be ‘presymptomatic’ for overt Rett-like signs [4], [5], [8]. Certain cellular and network features of MeCP2-deficiency, such as imbalance between cortical excitatory and inhibitory circuits, are apparent in *Mecp2^−/y^* mice from as early as 4–5 weeks [22], but many other features, such as diminished synaptic plasticity [13], [23] and the appearance of reduced locomotion, seizures and abnormal breathing, are typically reported to occur later in male *Mecp2* knockout mice [24].

Whilst RTT predominantly affects females, male knockout mice are typically used for RTT preclinical studies, in part to overcome the complexities resulting from mosaic expression of *Mecp2* in heterozygous females (due to X chromosome inactivation) but also because the course of phenotype development is more aggressive, more stereotyped and less variable in the males, and they therefore provide a more useful and experimentally tractable test-bed for putative therapeutic interventions [25].

The treadmill-based gait analysis used here has been validated in a variety of disease models including amyotrophic lateral sclerosis [26], Parkinson disease [27], [28] and Huntington disease [29], [30] and has been shown to be a simple, sensitive and objective method for detecting gait abnormalities. We have shown here that certain gait abnormalities can be detected very early in the disease progression in the RTT model used here, and we would argue that these measures should be evaluated as biomarkers for early and deep-seated aspects of the RTT-like phenotype, with a view to developing them as early indicators of amelioration and/or reversibility of RTT-like phenotypes in preclinical therapeutic studies [31]–[35].
